# The first case of neonatal priapism during hypothermia for hypoxic-ischemic encephalopathy and a literature review

**DOI:** 10.1186/s13052-018-0514-9

**Published:** 2018-07-27

**Authors:** Claudia Fanni, Maria Antonietta Marcialis, Maria Cristina Pintus, Cristina Loddo, Vassilios Fanos

**Affiliations:** Department of Surgical Science, University of Cagliari and Neonatal Intensive Care Unit, Puericulture Institute and Neonatal Section, Azienda Ospedaliera Universitaria, Cagliari, I-09042 Italy

**Keywords:** Newborn, Priapism, Neonatal priapism, Persistent penile erection, Cooling therapy

## Abstract

Neonatal priapism is a rare condition with only 26 described cases in literature since 1879. It is defined as a persistent penile erection occurring in the first 28 days of life, lasting at least 4 h that usually happens in the first days (from 2 to 12 days). It is a very different condition compared to the adult one because in newborns it is a relatively benign phenomenon. As a result of this paucity of described cases, classification and management are not well known by most of neonatologists and currently there are no established guidelines for its management. Most cases are idiopathic but other aetiologies are possible (polycythemia, blood transfusion and drugs). We describe our only case, which occurred during hypothermia therapy and review the literature to clarify the best choice in management of this rare entity.

## Background

The European Association of Urology guidelines define priapism as a disorder of penile erection, lasting more than 4 h, beyond or unrelated to sexual interest or stimulation [1, 2]. This refers to adults and children, while neonatal priapism is defined as a persistent penile erection occurring in the first 28 days of life, which usually happens in the first days (from 2 to 12 days) [3]. However neonatal priapism is not as well reported or understood in terms of aetiology, length of time and clinical aspects. Therefore the definition of neonatal priapism is incomplete because of the paucity of cases.

The term priapism refers to the Greek god *Priapus,* who was the first newborn suffering from this disorder. The myth says he was cursed by Hera when he was in the womb of his mother Aphrodite. Hera’s anger was triggered by Zeus’ affair with Aphrodite. Hera condemned the baby to ugliness, impotence and perversity. He was born with a grotesque aspect. The newborn had a giant nose, tongue, belly, feet, hands and in particular an oversized penis in a continuous state of erection [4, 5]. When Aphrodite saw him, she rejected the baby. Thus Priapus grew up with shepherds who quickly realised his magic effect on the environment because his presence was associated with thriving nature. Therefore he became the god of fertility and his phallus a symbol of power [6–8]. Even if the myth originated in Lampsacus (Anatolia), similar figures are present in different parts of the world and in different ages. However Priapus was always the protector of male genitalia and of fertility. Symbols representing him were set in lands to protect plants and animals and probably arrived in the present day in the form of our garden gnomes [4].

## Epidemiology

This disorder of penile erection may occur in all ages and its incidence in the general population is 0,5–0,9 cases per 100.000 persons-years [9, 10]. Evidence shows that priapism is extremely rare in newborns and the true incidence is unknown. Epidemiologic data on neonatal priapism is derived from only 25 cases reported in literature since 1876. Merlob and Livne found an incidence of 0,15 live births, in a surveillance study conducted between 1974 and 1988 [11].

## Physiology of erection

The states of erection and flaccidity depend on vascular condition. Corpora cavernosa are similar to a blood-filled sponge with irregular form, connected to each other and separated by trabeculae. Therefore the dilatation of helicine arteries leads to erection while the constriction of these arteries results in its flaccidity [12]. These vascular conditions are regulated by parasympathetic and sympathetic systems, both of which converge in the pelvic plexus. Nervous fibers of the sympathetic system arise in a portion of the spinal cord from the 12th thoracic to second lumbar segment, they come from the hypogastric plexus (immediately below the aortic bifurcation) and from here they go to the pelvic plexus. Nervous fibers of the parasympathetic system arise from the second to fourth sacral segments; both of them arrive in the pelvic plexus. From this plexus parasympathetic and sympathetic nerve fibers originate, which arrive in the corpora cavernosa. The activation of the parasympathetic system is responsible for the erection which takes place after a periferical (genital stimulation, reflexogenic pathway) or a central stimulus (thoughts, images). The activation of this system leads to the release of nitric oxide (NO) from cavernosal nerves and endothelial cells of helicine arteries in neuromuscular junctions. NO activates guanylate cyclase with the production of cyclic guanosine monophosfate (cGMP) from guanosine 5 triphosfate (GTP). The activation of the cGMP dependent protein kinase reduces the calcium concentration in the smooth muscle cells situated in the helicine artery walls. This determines the dilatation and the filling of the arteries [13]. Conversely, with the release of noradrenalin the sympathetic system causes an increase of intracellular calcium concentration, with consequent vasoconstriction of the helicine arteries [3].

## Classification and pathophysiology

In adults and children, priapism is commonly classified into three types: **ischemic, non ischemic and stuttering** [14].

**Ischemic priapism** (with little or no arterial flow) is the most common type (> 95%) of persistent erection in adults and children and the patients typically complain of penile pain. Ischemic priapism has been associated with many potential causative factors (Table 1). Most of these causes can lead to a veno occlusive condition with the congestion and slowing of the blood stream [15].

**Table 1:** Causes and physiopathology of ischemic priapism in older children and in adults

Cause	Percentage (%)	Physiopathology
Haemoglobinopathy (Sick cell disease)	65	Microvascular obstruction for sickling of deoxygenates Haemoglobin S in small vessel with low pO2 [3, 15].
Leukaemia	10	Impairment of vascular integrity with activation of protrombotic mechanism for interaction of leukaemic blasts and endothelial cells [3].
Tumor (primary or metastatic)	10	Can lead to priapism through direct infiltration and obstruction [17, 45–47].
Drugs: Erectile dysfunction pharmacotherapies Antihypertensives (hydralazine, prazosin) Antipsychotics (chlorpromazine) Antidepressants (trazodone) Alcohol Cocaine	5	Vasoactive mechanisms [1].
Infection and central disease	Not reported	Action on central erectile centre or failure in detumescence mechanism [17].
Haemodialysis	Not reported	Hypovolemia and haemoconcentration who results in increase of intravascular viscosity [17, 48].
Parental nutrition	Not reported	It contains fat emulsion because this situation causes a rise of intravascular viscosity, stimulates blood coagulability and also it is possible the development of fat embolism [17, 49, 50].
Toxins (scorpion, spider)	Not reported	Action in Calcium and Potassium channels on the vascular smooth muscle cells [51].
Henoch –Schonlein purpura	Not reported	Obstruction to venous outflow [52].

In 10% the cause remains idiopathic, but on the basis of recent studies a possible role is related to platelet activity, which can contribute to veno-occlusion [16]. In all these cases there is venous congestion and a rise of blood viscosity [17].

### Clinical and ultrasound findings of ischemic priapism (with little or no arterial flow)

The erection is always painful. In a physical examination the patient presents stiffness of the corpora cavernosa and a flaccid and spongiosum gland [3]. The main finding in Colour Duplex Ultrasonography (CDU) is the absence of cavernous blood flow with a “high resistance, low velocity” wave form [3, 18]. Blood gas analysis of corpora cavernosa shows dark and deoxygenated blood (pO2 < 40 mmHg) [3]. In this type of priapism a compartment syndrome can develop after 4 h of prolonged erection. This phenomenon could become an urological emergency due to the fibrosis of the cavernous bodies consequent to hypoxia [1, 2].

### Non ischemic priapism

It is caused by a blunt perineal trauma which results in a rupture of a cavernosal artery. Consequently there is a formation of a high flow fistula between an arteriole and lacunar spaces of the sinusoidal tissue [1]. The arteriole rupture, usually in the crura or in corporal bodies is consequent to a penile, perineal, pelvic trauma [3]. More rarely the fistula can be iatrogenic after an intracavernosal injection or aspiration [1].

### Clinical and ultrasound findings of ischemic priapism (arterial or high flow)

The erection is painless. The physical examination shows a not fully rigid erection, and it is possible to observe sign of the trauma at perineal or abdominal level [1, 2].

CDU shows low resistance, a high flow arterial waveform and the arterio-sinusoidal fistulae can be seen [3, 18]. In this case, the blood gas analysis shows oxygenated blood with a pO2 > 90 mmHg [3].

In the **Stuttering** form, priapism is recurrent or intermittent [1].

This form is very common in children and adults affected by sickle cell disease or it could be related to a neurological problem or idiopathic [1].

The aetiology and pathogenesis is similar to that of a milder ischemic form [3].

### Neonatal priapism

A transient erection in healthy newborns is very common. A physiological erection lasting a few minutes could be spontaneous [19] or consequent to different stimulation (tactile, diaper changing, bathing, urethral catheterization, full bladder) [20] and demonstrates the normal function of the nerve to the penis [21]. Conversely, a persistent penile erection lasts hours but a well defined definition is not reported in literature. Donaldson was the first author who considered the neonatal form of persistent penile erection as a fourth type of priapism. In fact, neonatal priapism cannot be considered an ischemic form because of the normal CDU and absence of pain and fibrosis. Neither can it be considered a non ischemic form due to the normal CDU. Neonatal priapism is more frequently related to polycythemia [22–24]. Literature reports other associations such as blood transfusions [25], infections (congenital syphilis [26], pyocavernositis [27]), cranial birth traumas (forceps), respiratory distress syndrome, umbilical artery catheterization [3], neurological pathologies, drug–related side effects [28] and parenteral nutrition [22]. Differently from what happens in older children, due to the predominance of foetal haemoglobin, SCD is not a cause of priapism in newborns [21]. However, in the majority of cases the aetiology remains idiopathic [19–21, 29–37].

## Case report

**Fig. 1:**
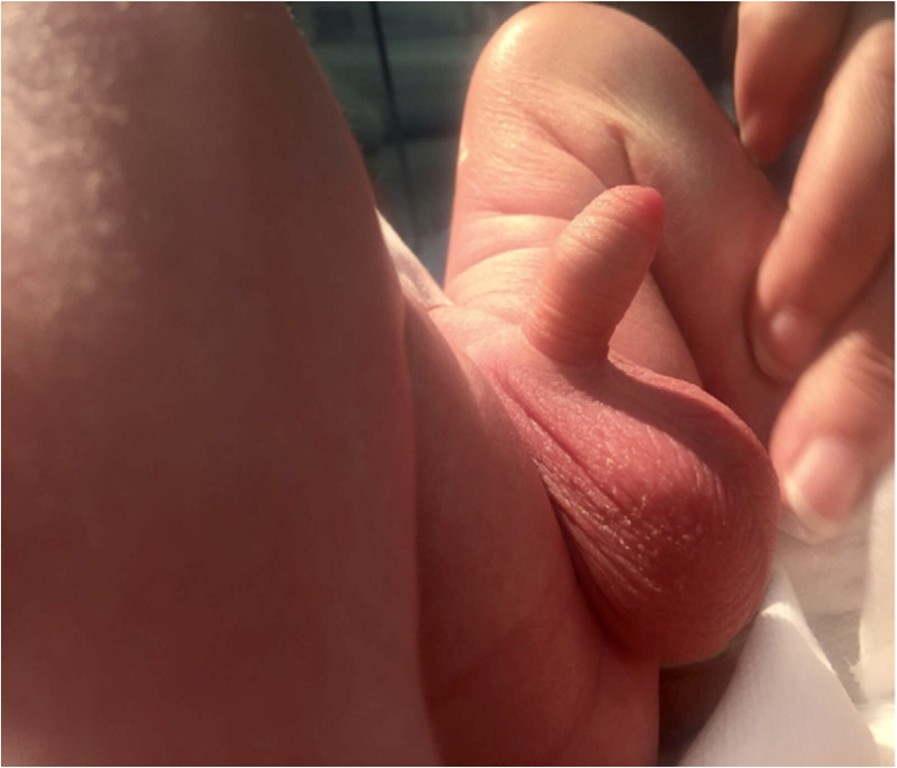
Neonatal priapism: neonatal priapism in our newborn

**Fig. 2:**
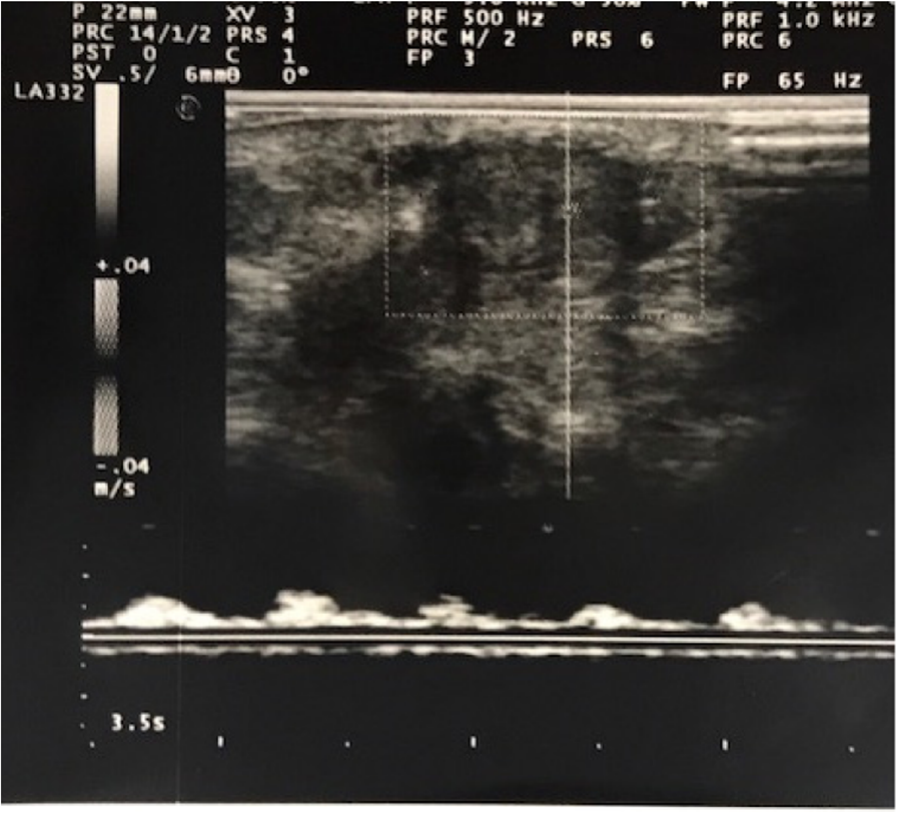
Color Doppler ultraonography *Normal arterial flow on Colour Doppler Ultrasonography (CDU) of the penis in our newborn with priapism*

Our baby was born at 40 weeks and 2 days with a birth weight of 3380 g, to a 37-year-old healthy woman with an uneventful pregnancy. A Caesarean section was performed because of foetal distress and the Apgar score was 5, 7 and 7 at 1, 5 and 10 min respectively. The infant required cardio-pulmonary resuscitation at the first minute and than he was transferred to our Neonatal Intensive Care Unit. He was admitted to our Department with a diagnosis of moderate hypoxic ischemic encephalopathy. Consequently he was submitted to hypothermia treatment at 4 h and immediately he showed a persistent painless penile erection (Fig. 1) without discolouration of the scrotum or penis, and with bilaterally palpable testicles. An Ultrasonography examination of the arteries and the veins of the penis (Fig. 2), and laboratory findings (blood count and biochemical parameters) were normal. Once confirmed non-ischemic priapism, conservative treatment was chosen. Detumescence occurred after 4 h. The newborn continued to have intermittent erections with minimal stimulation for three days. At discharge physical examination was normal. On follow-up at 15 days and at one month, the patient had a normal physical examination and the mother reported normal erections.

## Literature review

Below we discuss the principal features of neonatal priapism cases found on Medline from 1974 [23] to 2018, using priapism, newborn, neonatal priapism, persistent penile erection as key words.

Until today 26 cases of neonatal priapism have been reported and all cases are presented in Table 2.

**Table 2:** Date of 27 cases [Rearranged with additional 6 cases from reference Talibzade]

Reference	Onset	Treatment	Duration (days)	Proposed aetiology
Nuckols [26]	Not reported	Not reported	Not reported	Not reported
Humbert et al. [22]	Day 1	Observation	2	Polycythemia
	Day 4	Phlebotomy	5	Polycythemia
Laroque and Cosgrove [23]	Not reported	Observation	4	Polycythemia
Amlie et al. [25]	Day 37 (delivery at 29 weeks)	Observation	12	Blood transfusion
Leal et al. [29]	At birth	Observation	6	Idiopathic
Shapiro [30]	At birth	Observation	3	Idiopathic
Merlob and Livne [11]	At birth	Observation	6	Idiopathic
	Day 1	Observation	5	Idiopathic
	Day 5	Observation	4	Idiopathic
	Day 1	Observation		Idiopathic
Stothers and Rictchie [31]	At birth	Intravenous ketamine	3	Idiopathic
Walker and Casale [24]	At birth	Exchange transfusion	4	Polycythemia
Meijer and Bakker [32]	Day 1	Observation	4	Idiopathic
Burgu et al. [21]	Day 1	Observation	3	Idiopathic
Aktoz et al. [20]	Day 1	Observation	4	Idiopathic
Sood et al. [27]	Day 4	Surgery	20	Pyocavernositis
Dust et al. [33]	Day 1	Observation	6	Idiopathic
Karakaja et al. [36]	Day 7	Observation	3	Idiopathic
Marmara [34]	Day 1	Observation	6	Idiopathic
	Day 1	Observation	7	Idiopathic
Kuwano et al. [19]	At birth	Observation	5	Idiopathic
Laamiri et al. [37]	Day 1	Observation	9	Idiopathic
Miller and Roth [28]	–	Observation	3	iNO therapy, Sildenafil
	–	Observation	–	Sildenafil
Samiee S. et al. [35]	Day one	Observation	5	Idiopathic
Present case	Day one	Observation	3	Idiopathic

The onset of penile erection varied from 1 to 37 days of life. The duration of the phenomenon was variable from 2 to 12 days. Most of the published cases showed the same clinical features and were included in the non-ischemic form with normal CDU. Only one case differed from the others because it was complicated by pyocavernositis. In the majority of the cases the erection was painless and without discolouration of the penis or scrotum. In most of the cases reported in literature a simple close observation was carried out and a spontaneous resolution without short-term sequelae was reported (maximum follow-up period of 20 months) [32]. The first case of priapism, in 1876, was described in a newborn with congenital syphilis but the duration, management and follow-up were not reported [26]. Only ninety-three years later, other authors started to describe consecutive and detailed cases of persistent penile erection in newborns. In the majority of cases the aetiology is unknown [11, 19–21, 29–37]. However, in four of the reported cases, priapism was associated with polycythemia [22, 24]. In another one, priapism followed a blood transfusion [25]. In addition, Sood et al. [27] described a very singular case of priapism occurring in the fourth day of life during spontaneous pyocavernositis caused by *Klebsiella spp*. The baby suffered from fever and his penis was warm and erythematosus with a hump at the base. Laboratory testing was normal but the aspiration drainage, performed under general anaesthesia, revealed frank pus. The case recovered after aspiration drainage and irrigation with local and systemic antibiotics. Finally, in 2005, two drug-related cases were reported. The first persistent penile erection happened during treatment with inhaled nitric oxide (iNO) and resolved itself 10 min after the withdrawal of the therapy. The second drug-related case occurred one hour after starting oral Sildenafil therapy for Primary Pulmonary Hypertension (PPH) and persisted for the whole duration of the therapy [28]. The last case of priapism classified as idiopathic was found associated with transient bladder retention or an inflammatory pseudotumor of the bladder, related to a catheterization for decreasing urinary output [35]. In only two reported cases, management consisted of phlebotomy and exchange transfusion [22, 24]. The use of intravenous ketamine was reported in one case [31]. In most cases blood gas analysis was not performed and done only after a CDU revealed an ischemic form [19, 33].

## Discussion

According to the published data, persistent penile erection in neonates should be separated from the other forms of priapism affecting older children and adults because it is generally benign and requires observation alone. Since the literature showed only individual case reports and the definition of neonatal priapism is still inconsistent, a consensus document has not been developed yet. Priapism in newborns is generally idiopathic. Neonatologists should consider different elements when collating the history of priapism. According to literature the most important factor correlated with priapism is polycythemia. The underlined mechanism could consist in increased blood viscosity which may hinder the outflow of blood from the penis and cause thrombosis [22–24]. In 2 out of 26 cases there was a temporal correlation between priapism and both iNO therapy and oral Sildenafil. Since NO is an important factor in the genesis of the physiological erection, persistent penile erection could be explained by the increased concentrations of iNO during therapy. According to the authors, Sildenafil, a phosphodiesterase type-5 inhibitor, could promote the erection by enhancing the effect of nitric oxide [28]. In one case, priapism followed a blood transfusion [25]. The mechanism hypothesized in this case is related to vascular constriction, consequent to the hypoxic condition. In addition banked red blood cells may be less competent to increase oxygen delivery to the penis leading to ischemia and vasoconstriction with consequent stasis in venous plexus [25, 32]. Our case is the first showing priapism during hypothermia treatment for hypoxic ischemic encephalopathy (HIE). The correlation between hypothermia and priapism is unknown. In our opinion, several physiologic responses to cooling therapy may trigger neonatal priapism. It is common knowledge that hypothermia implies changes in the dynamic of the circulatory system. The low temperature causes a centralization of the circulation with a redistribution of cardiac blood flow to the cerebral compartment [38]. Thus the body of the newborn tries to conserve heat through vasoconstriction [39]. In addition, a decrease in intravascular volume due to fluid shifts and cold-induced diuresis is reported. Finally in vitro studies showed a decreased deformability of blood cells and a rise of blood viscosity during cooling therapy [40–42]. Furthermore during asphyxia a rise in the synthesis of NO is reported [43], which is the most important mediator of priapism. Moreover, during the first three days following asphyxia, nitric oxide concentration in both serum and cerebrospinal fluid is higher than normal. All of these factors could contribute to the onset of priapism. Differently from adults, neonatal priapism generally shows spontaneous resolution and requires careful observation with conservative management [3]. In the majority of cases detumescence is spontaneous. First-line investigations should include careful assessment to rule out a common cause of prolonged erections in neonates such as polycythemia or a full bladder. Blood count, C-reactive protein [3] and drug related aetiology need to be evaluated. Due to the favourable natural history of priapism in the majority of cases, blood gas analysis should not be performed. Bacterial infection and blood transfusion related reactions should be excluded. Careful clinical examination should exclude the presence of pain or local inflammatory and traumatic signs of the pelvis, perineum or penis, discolouration of skin, and a full bladder. Minimally invasive diagnostics (CDU) should be performed. The non-invasiveness of CDU makes it suitable and safe in newborns. Therapeutic aspirations and exchange-transfusion do not seem to be effective while intravenous ketamine could be dangerous in particular in newborns where the development of the immature brain is rapid and different publications underline its possible role in neuroapoptosis and synaptogenesis [44]. On the basis of careful analysis of the literature we decided on conservative management with close observation and a Doppler ultrasound of the penis.

## Conclusions

Neonatal priapism should not generate anxiety in neonatologists because in the majority of cases it is a benign condition. As a result of this paucity of described cases, classification and management is not well known and currently established guidelines for its management do not exist. Even if prolonged erection in neonates does not seem to be associated with any impairment of sexual function, further studies are needed to clarify if these beliefs are related to a lack of long-term follow up.

## Abbreviations

cGMP: cyclic guanosine monophosfate
GTP: Guanosine 5 triphosfate
i NO: inhaled nitric oxide
NO: Nitric oxide
PPH: Primary pulmonary hypertension
